# Surface Acoustic Wave (SAW) Resonators for Monitoring Conditioning Film Formation

**DOI:** 10.3390/s150511873

**Published:** 2015-05-21

**Authors:** Siegfried Hohmann, Svea Kögel, Yvonne Brunner, Barbara Schmieg, Christina Ewald, Frank Kirschhöfer, Gerald Brenner-Weiß, Kerstin Länge

**Affiliations:** 1 Institute of Microstructure Technology, Karlsruhe Institute of Technology, Hermann-von-Helmholtz-Platz 1, 76344 Eggenstein-Leopoldshafen, Germany; E-Mails: siegfried.hohmann@partner.kit.edu (S.H.); svea.koegel@kit.edu (S.K.); ybrunner@stud.hs-offenburg.de (Y.B.); barbara.schmieg@kit.edu (B.S.); christina.ewald@partner.kit.edu (C.E.); 2 Institute of Functional Interfaces, Karlsruhe Institute of Technology, Hermann-von-Helmholtz-Platz 1, 76344 Eggenstein-Leopoldshafen, Germany; E-Mails: frank.kirschhoefer@kit.edu (F.K.); gerald.brenner-weiss@kit.edu (G.B.-W.)

**Keywords:** surface acoustic wave (SAW), two-port resonator, conditioning film, human serum albumin (HSA), fibrinogen, plasma protein, implants, quartz crystal microbalance (QCM-D), polymer

## Abstract

We propose surface acoustic wave (SAW) resonators as a complementary tool for conditioning film monitoring. Conditioning films are formed by adsorption of inorganic and organic substances on a substrate the moment this substrate comes into contact with a liquid phase. In the case of implant insertion, for instance, initial protein adsorption is required to start wound healing, but it will also trigger immune reactions leading to inflammatory responses. The control of the initial protein adsorption would allow to promote the healing process and to suppress adverse immune reactions. Methods to investigate these adsorption processes are available, but it remains difficult to translate measurement results into actual protein binding events. Biosensor transducers allow user-friendly investigation of protein adsorption on different surfaces. The combination of several transduction principles leads to complementary results, allowing a more comprehensive characterization of the adsorbing layer. We introduce SAW resonators as a novel complementary tool for time-resolved conditioning film monitoring. SAW resonators were coated with polymers. The adsorption of the plasma proteins human serum albumin (HSA) and fibrinogen onto the polymer-coated surfaces were monitored. Frequency results were compared with quartz crystal microbalance (QCM) sensor measurements, which confirmed the suitability of the SAW resonators for this application.

## 1. Introduction

Biofilms are aggregates of microorganisms that can be found ubiquitously at interfaces as long a sufficient amount of humidity is provided. They occur in a wide variety of manifestations, ranging from beneficial biofilms in biotechnological processes to harmful biofilms in technical systems leading to biofouling. Though biofilms are a common phenomenon, they are not yet fully controllable and therefore still a topic of investigation [1,2,3]. Biofilm-related research includes the topic of conditioning films. The moment an interface is formed, for instance, when a substratum comes into contact with an aqueous fluid, inorganic, organic, and macromolecular substances will adsorb on the substratum surface forming the conditioning film. Such a film will alter the physico-chemical properties of the substrate surface. Furthermore, conditioning films comprising proteins may offer additional receptor sites for microorganisms to bind. As a result, initial conditioning layers greatly influence the adherence of subsequently adsorbing microorganisms, whereas conditioning film formation itself strongly depends on the underlying substratum material [4,5,6,7,8]. Therefore, it is necessary that methods investigating conditioning film formation allow the application of a variety of coatings, representing the respective substrata. Furthermore, time-resolved monitoring of the molecular deposition, as well as a fluidic handling system for *in situ* measurements, would be advantageous. 

As depicted above, proteinaceous films are of special interest as they may add biological functionality to the substratum. In the first step of implant surgery, for instance, blood proteins adsorb spontaneously on the implant surface. This starts the wound healing process, but also adverse immune reactions leading to inflammatory responses. Hence, a means to promote the former and suppress the latter would be the control of the initial protein adsorption [4,5,9,10,11]. The most abundant blood protein at a concentration of 35–53 mg/mL is human serum albumin (HSA). Physiological conditions provided, HSA is an approximately globular (“heart-shaped”) protein with a molecular weight (MW) of 66 kDa. Albumin adsorption typically results in dense single layers. Another important blood protein is fibrinogen. Occurring at plasma concentrations in the range of 1.5–4.5 mg/mL it is the most abundant plasma protein taking part in the coagulation cascade. Fibrinogen is described as an elongated molecule with a MW of 340 kDa. The elongated structure allows more variations in the molecular orientation on the surface than a globular structure as found, e.g., for HSA [12,13,14,15,16,17,18]. However, protein adsorption depends not only on the protein itself but also on external parameters, such as physical and chemical properties of the substrate surface, as well as composition (including pH) of the surrounding medium. Therefore, in most cases, results obtained with a specific protein (or protein mixture) on a specific surface in a specific medium cannot be readily transferred to another protein (mixture) or surface or medium. Instead, it is necessary to investigate protein adsorption on the surface and the medium in question individually [17,18,19].

User-friendly methods for time-resolved monitoring of protein adsorption on substrata are readily available as detectors used in biosensor setups. Biosensors are integrated receptor-transducer devices used to detect a variety of analytes, including proteins. Furthermore, biosensor setups typically are accompanied by a fluidic system allowing the controlled handling of liquid samples. For protein adsorption in terms of conditioning film monitoring on a specific substrate, the receptor coating of the transducer would be redundant; instead, a coating with substratum material will be required. Most commonly used biosensor detectors are based on electrochemical, optical, and acoustic transduction principles [20,21]. In the case of conditional film monitoring, optical and particularly acoustic transducers enable a greater variety of coatings, which do not interfere with the transduction principle than electrochemical transducers. Hence, sensor setups reported for *in situ* proteinaceous conditioning film monitoring, thus far, are mainly based on surface plasmon resonance (SPR) and quartz crystal microbalance (QCM) [10,13,22,23]. Regarding the evaluation of sensor signals, it has to be considered that they usually result from a sum of events on the surface, such as molecular binding or conformation change. Consequently, such a sensor signal cannot be correlated with a single type of event only. As different sensors respond to different effects, a combination of several transduction principles should result in complementary information, allowing a more comprehensive characterization of the adsorbing protein layer. This has been shown, for instance, by combining QCM with optical waveguide lightmode spectroscopy (OWLS) and ellipsometry and by combining SPR, QCM, surface acoustic wave (SAW), and atomic force microscopy (AFM) for time-resolved *in situ* investigation of protein adsorption on Teflon AF, TiO_2_, and hydrophobized gold [14,24,25]. 

QCM sensor setups are widely available as QCM-D setups, *i.e.*, QCM with dissipation monitoring. This setup allows two independent signal readouts at the same time: frequency, which is linked to the mass of the adsorbed layer, and dissipation, which is linked to the viscoelastic properties of the layer and, thus, with the conformation of the adsorbed molecules included [26,27]. Similar to that, SAW sensor setups are available which read out both phase, which is mainly linked to the layer mass, and amplitude, which is linked to the layer viscoelasticity. The acoustic energy of SAW sensors is strongly confined at the surface of the devices. Therefore, the SAW is potentially very sensitive towards changing influences on the surface, such as mass loading as well as changes in viscosity and viscoelasticity [28,29,30,31]. SAW sensor frequencies are also more susceptible to temperature changes than QCM sensor frequencies, because the temperature coefficients of frequency (TCF) of the SAW sensor substrates (e.g., ST-cut quartz, LiTaO_3_, LiNbO_3_) are higher than the TCF of AT-cut quartz, which is typically used as QCM sensor substrate. However, this can be overcome by additional SiO_2_ layers, which reduce the TCF, or by an appropriate external thermostatic control [26,32,33,34]. Furthermore, resonance frequencies of SAW devices are usually higher than those of QCMs. This is particularly advantageous, because the mass sensitivity increases with increasing resonance frequency. Finally, being commonly produced using lithography and subsequent metal deposition, SAW sensor devices are compatible to mass production which typically minimizes production costs. SAW devices have proven to be suitable as cheap and disposable sensor elements. SAW sensor arrays guiding liquid samples across a set of different surfaces have recently been introduced [31,35,36,37].

**Figure 1:**
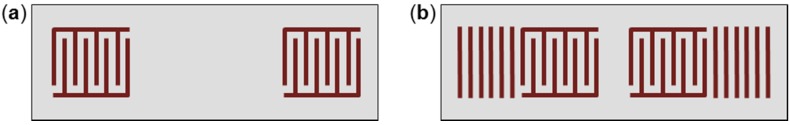
SAW sensor configurations (**a**) delay line (**b**) resonator (two-port).

Most SAW devices presented to the scientific community are delay line devices (Figure 1a). Since the SAW has to travel a comparatively long distance on the surface of these devices, which is usually equipped with sealing elements to keep the transducers separated from passing fluids, uncoated delay line devices usually suffer from high insertion losses. Therefore, these devices are often coated with a wave-guiding layer to obtain Love mode devices with decreased insertion loss. As delay line devices do not feature a single defined resonance frequency, they are typically evaluated by detection of phase and amplitude shift, as mentioned before, which requires a more complex electronic setup. SAW resonators (Figure 1b), on the other hand, produce surface waves similar to surface transverse waves (STW), because the reflective fingers act as a mass grating, which guides the SAW on the substrate surface. The resonator design typically requires complete immersion in the liquid channel, including the transducers. Still, compared to a classical delay line setup, SAW resonators feature smaller insertion losses and, hence, can be used without additional wave-guiding layers. Furthermore, SAW resonators provide very distinct and sharp resonances. Therefore, the detection of the resonance frequency is easily achievable with simple and economical electronic setups, such as oscillators [28,31,38].

In this work, SAW resonators have been used for monitoring plasma protein adsorption—as part of the proteinaceous conditioning film formation—on polymers. Plasma proteins were HSA and fibrinogen. Polymers were parylene C (poly(2-chloro-p-xylylene)), polymethyl methacrylate (PMMA), and polystyrene (PS). These polymers served as examples for an implant coating material (parylene C), for components of intraocular lenses, dental fillings, and bone cement (PMMA), as well as standard surfaces for protein adsorption studies (PS) [13,39,40,41,42,43]. In the first part of this work, the adsorption of HSA and fibrinogen was monitored with parylene C coated SAW resonators. The frequency responses were compared with those obtained with parylene C coated QCM sensors to show that additional information was obtained using another acoustic sensor. Furthermore, SAW resonators were coated with PMMA and PS for use in HSA adsorption measurements to show the general suitability of SAW resonators for monitoring proteinaceous conditioning film formation on polymers. 

## 2. Experimental Section 

### 2.1. SAW Resonator (Sensor) Measurement Setup

**Figure 2:**
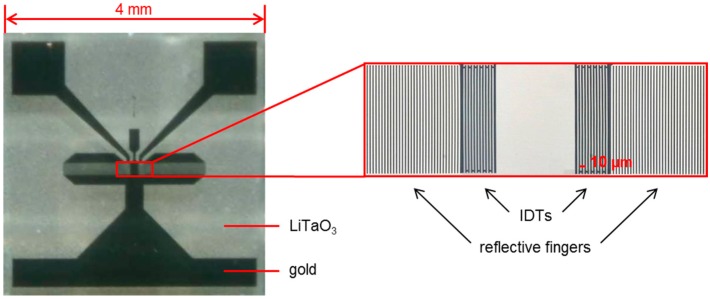
SAW resonator type SR062 consisting of LiTaO_3_ substrate with gold transducers.

Shear horizontal SAW resonators type SR062 were delivered by SAW Components, Dresden, Germany. The resonators were based on small (4 mm × 4 mm) 36°YX-LiTaO_3_ devices with gold transducers (Figure 2). The acoustic aperture of the IDTs was 0.2 mm. The frequency of operation determined in PBS was 426.4 MHz. SAW measurements were performed in an oscillator circuit developed in-house with the SAW resonator sensor as frequency-determining element as described earlier [44]. The phase was set by selecting an appropriate drive voltage via a capacity diode. Resonator frequencies were determined as difference frequencies relative to a reference resonator oscillating permanently at 433.9 MHz while the phase was kept constant. The frequency resolution was 1 Hz. For the sake of clarity, SAW resonator measurements were plotted to start at 0 Hz instead of starting at the actual difference frequency. 

**Figure 3:**
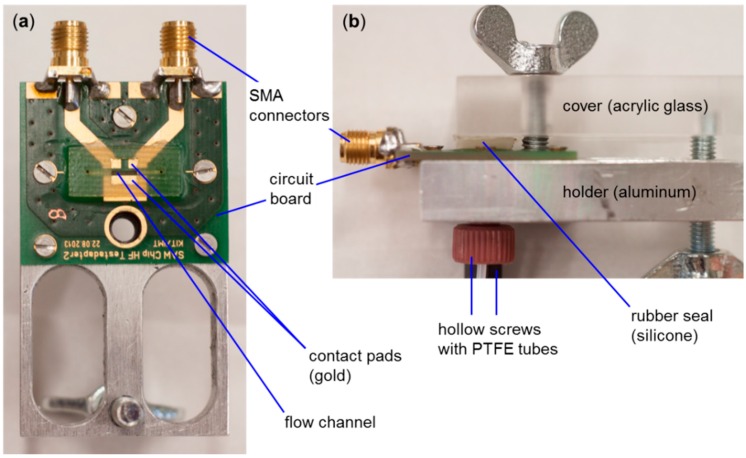
Flow cell connecting the SAW resonator to the driving electronics and the peripheral fluidic system. (**a**) Open flow cell (top view), without cover; (**b**) Flow cell closed with cover (side view).

A flow cell was designed (Figure 3) in which the SAW resonator device was mounted, face down, onto isolated contact pads on the electronic board and coupled capacitively to the driving electronics. The circuit board and driving electronics were interfaced by two standard SMA connectors for input and output signals. A flow channel in between the contact pads allowed guiding liquids along the active structure of the SAW resonator (Figure 3a). Channel dimensions were 4 mm (length) × 0.601 mm (width) × 0.569 mm (depth), resulting in an effective sample volume above the sensor of 1.4 µL. The SAW resonator is sealed by closing the flow cell’s cover providing a rubber seal. Hollow screws guiding polytetrafluoroethylene (PTFE) tubes were used as connections to the fluidics (Figure 3b).

**Figure 4:**
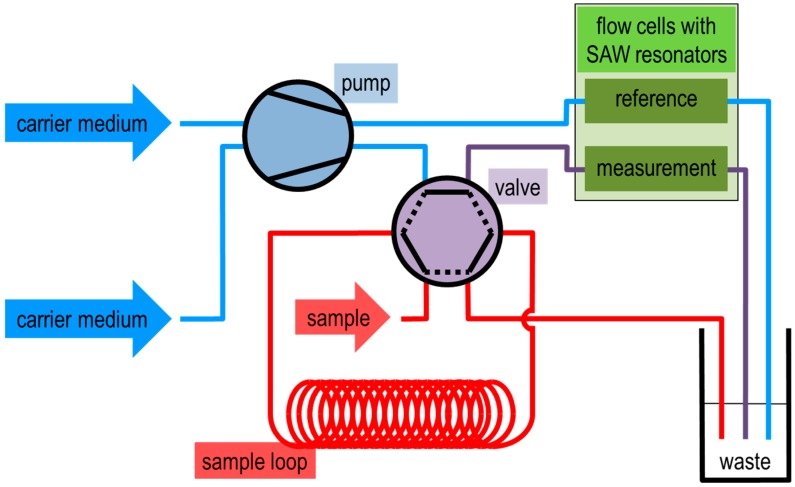
Flow injection analysis system for the SAW resonator measurement setup. Carrier medium was driven by a pump through the reference flow cell or through a valve connected with the measurement flow cell. The solid lines in the valve represent the load mode, in which the sample loop is loaded while the measurement cell is rinsed with carrier medium. The dotted lines represent the inject mode in which the carrier medium moves the sample through the measurement cell.

Protein binding experiments were performed by means of a flow injection analysis (FIA) system, as depicted in Figure 4. The FIA system was equipped with a two-channel peristaltic pump (Ismatec, Wertheim, Germany), a six-port sample injection valve (Besta-Technik, Wilhelmsfeld, Germany), and two flow cells with integrated SAW resonator devices. One flow cell was used for the protein binding measurements (Figure 4, flow cell “measurement”), the other was sampled with carrier medium only and served as reference (Figure 4, flow cell “reference”). PTFE tubes served as sample loop, *V* = 5 mL, and as connections between single components. The injection valve allowed switching between load and inject modes. In the load mode (Figure 4, valve mode “solid lines”), carrier medium (buffer) was driven by a pump through the measurement cell, while the sample loop was loaded with sample (protein solution) by means of a syringe. During the inject mode (Figure 4, valve mode “dotted lines”), the content of the sample loop was moved by the carrier medium through the measurement cell and, hence, across the SAW resonator. 

Additionally, for protein adsorption tests on various polymers a similar FIA system as shown in Figure 4 was applied, but with a smaller sample loop (*V* = 0.2 mL) and without a reference cell.

### 2.2. QCM Sensor Measurement Setup

QCM sensors were based on AT-cut quartz crystals (diameter: 14 mm), sandwiched between a pair of gold electrodes, and a frequency of operation of 4.95 MHz (type QSX 301, Q-Sense). QCM sensor measurements were performed with a commercial QCM-D instrument, Q-Sense E4, which was purchased from LOT-Oriel, Darmstadt, Germany. Resonance frequencies and dissipation shown in the following were recorded at the third overtone. For the sake of clarity, QCM sensor measurements were plotted to start at 0 Hz instead of starting at the actual resonance frequency. 

**Figure 5:**
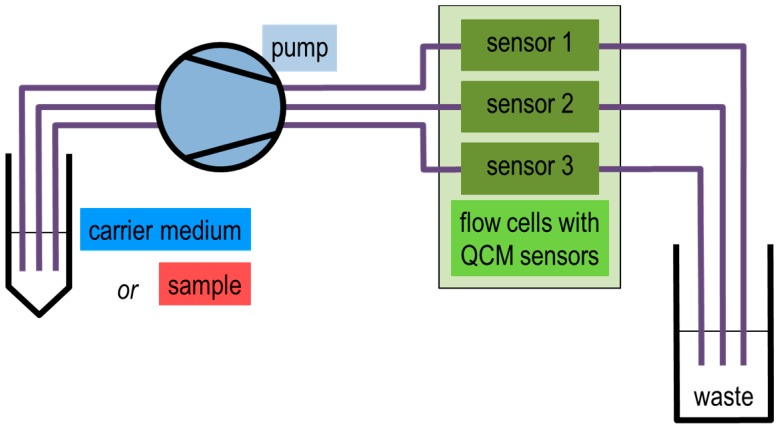
Flow system used for the QCM sensor measurement setup. Either carrier medium or sample was driven by a pump through the flow cells.

Protein binding experiments were performed by means of a flow system as depicted in Figure 5. Three flow cells (flow modules type QFM 401, Q-Sense) were used in parallel, each one containing a QCM sensor. The effective sample volume above each sensor was specified as ~40 µL by the manufacturer. PTFE tubes served as connections between single components. Solutions were driven by an external four-channel peristaltic pump (Ismatec, Wertheim, Germany) through the flow cells and, hence, across the sensors (Figure 5, flow cells “sensor 1/2/3”). To switch between carrier medium (buffer) and sample (protein solution), the pump was stopped and the inlet tubes put in the respective reservoir.

### 2.3. Polymer Coatings and Contact Angle Measurements

SAW resonator sensors and QCM sensors were coated with 0.1 µm parylene C by chemical vapor deposition (CVD) using a commercial parylene deposition system (type Labcoater 1, PDS 2010; purchased from Specialty Coating Systems, Indianapolis, IN, USA). Within this process, parylene C dimer (di(2-chloro-p-xylylene)) was sublimated and subsequently pyrolyzed at 690 °C. The resulting monomer spontaneously polymerizes on the sensor surfaces provided in a vacuum chamber at room temperature forming solvent-free thin films of high packing density and low internal stresses. The process has earlier been described in detail [45,46].

Polymethyl methacrylate (PMMA, *M_r_* 550,000; purchased from Alfa Aesar, Karlsruhe, Germany) and polystyrene (PS, multiwell plate lids; purchased from VWR, Bruchsal, Germany) coatings of SAW resonators were made by using a spin coater (type WS-400-6NPP-LITE; purchased from Laurell, North Wales, PA, USA) and toluene solutions of the respective polymer. Parameters for the spin coating procedure are summarized in Table 1. To allow remaining solvent residues to evaporate, spin coated SAW resonators were used for protein adsorption experiments not until the next day. 

**Table 1:** Spin coating parameters for PMMA and PS layers.

Spin Coating Parameter	PMMA	PS
Polymer concentration in toluene [mg/mL]	0.1	10
Rotation speed [rpm]	5000	1500
Rotation time [min]	3	2
Acceleration step [a. u.]	10 (highest)	10 (highest)

Contact angle measurements were performed by the sessile drop method using a contact angle measurement microscope (type 20668; purchased from Erma, Tokyo, Japan).

### 2.4. Protein Adsorption Measurements for Conditioning Film Monitoring

Carrier medium was phosphate buffered saline (PBS), pH 7.4, ionic strength 0.15 M, representing physiological conditions. PBS was prepared from tablets (Sigma-Aldrich, Taufkirchen, Germany), which were dissolved in distilled water according to the manufacturer’s instructions. Protein samples contained HSA, fraction V, MW 66 kDa (VWR, Bruchsal, Germany), or fibrinogen from human plasma, MW 340 kDa (Sigma-Aldrich, Taufkirchen, Germany). The proteins were dissolved in the carrier medium at a concentration of 250 µg/mL or 1 mg/mL. 

Measurements were performed at room temperature. The polymer-coated sensors were inserted in the flow cells of the respective measurement setups and rinsed with carrier medium. The flow rate was set to 0.05 mL/min. The continuous flow ensured a constant temperature in the flow cells and, hence, of the sensors. When a stable baseline signal was obtained, the protein sample was applied on the sensor surface by the respective fluidic system, as described above. The SAW resonator measurement setup was operated with a sample injection interval of 1–76 min (5 mL sample loop) or 1–5 min (0.2 mL sample loop). The QCM sensor measurement setup was operated with a sampling interval of 3–103 min. Each protein adsorption experiment was performed with a separate sensor. In between the measurements, the PTFE tubings were thoroughly rinsed with distilled water, 1 M hydrochloric acid (Sigma-Aldrich, Taufkirchen, Germany), and 1% (*v*/*v*) Hellmanex II (Hellma Analytics, Müllheim, Germany) solution. 

## 3. Results and Discussion

### 3.1. Conditioning Film Formation on Parylene C: Adsorption of Plasma Proteins 

**Figure 6:**
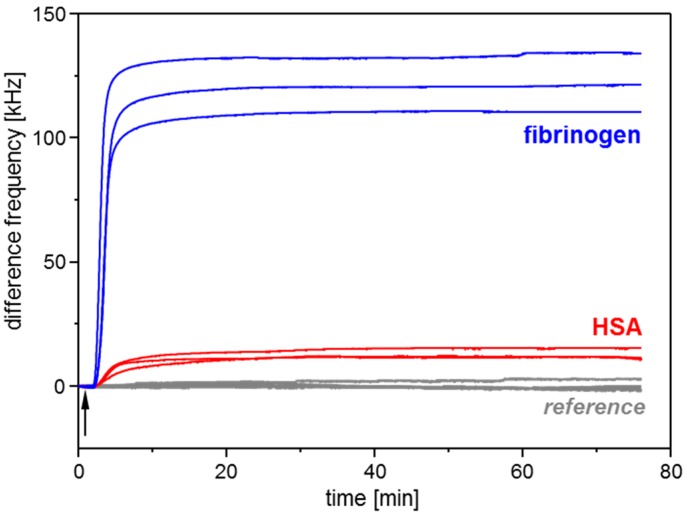
Conditioning film monitoring with SAW resonators coated with parylene C: Adsorption of plasma proteins HSA (red curves) and fibrinogen (blue curves). Samples contained 250 µg/mL protein in PBS and were injected into a PBS carrier stream. Injection started 1 min after start of the measurement (see arrow). Gray curves represent the signals obtained with the reference resonators, which were rinsed with carrier medium PBS throughout the complete measurement.

**Figure 7:**
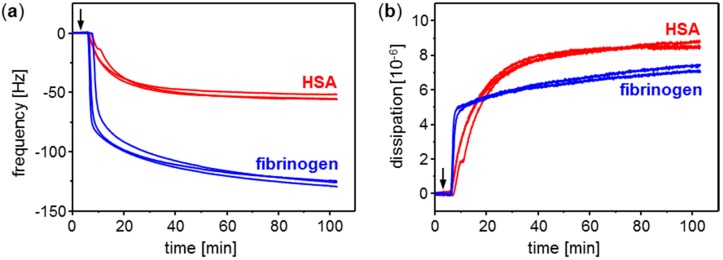
Conditioning film monitoring with QCM sensors coated with parylene C: Adsorption of plasma proteins HSA (red curves) and fibrinogen (blue curves). Samples contained 250 µg/mL protein in PBS. The PBS carrier stream was switched to sample solution 3 min after start of the measurement (see arrow). (**a**) Frequency; (**b**) Dissipation.

The adsorption of the plasma proteins HSA and fibrinogen on parylene C was monitored with parylene C coated SAW resonator sensors (Figure 6) as well as with parylene C coated QCM sensors (Figure 7). The SAW resonators and the QCM sensors showed opposite frequency signal responses resulting from protein adsorbing on the sensor surface. This contrast is mainly based on the difference in the signal output: The mass increase on the acoustic sensors’ surface leads to a decrease of the resonance frequency. This is directly shown by the QCM sensor signals (Figure 7a). As the SAW resonator frequencies are determined as difference frequencies relative to a reference resonator oscillating at a higher frequency (see Section 2.1), the mass increase here results in increasing difference frequencies (Figure 6, red and blue curves). The reference resonators included in the SAW resonator measurement setup were in contact with carrier medium PBS only. They showed no signal change linked to sample injection (Figure 6, gray curves), which confirmed that the signal increase observed with the measurement resonators resulted from adsorption of proteins present in the respective samples. 

Table 2 summarizes frequency shifts obtained with parylene C coated SAW resonators and QCM sensors after adsorption of the plasma proteins HSA and fibrinogen in PBS (see Figure 6 and Figure 7a). The frequency shifts were determined as mean frequency in the last minute of the respective adsorption interval. Furthermore, in the time interval prior to fibrinogen adsorption, the noise amplitudes of the sensors were determined. With the frequency shift obtained by protein adsorption set as signal amplitude, the respective signal-to-noise ratio (S/N ratio) was calculated as ratio of signal amplitude to noise amplitude. HSA adsorption on parylene C coated sensors resulted in S/N ratios of 158 (SAW resonator) and 169 (QCM sensor); fibrinogen adsorption resulted in S/N ratios of 1524 (SAW resonator) and 396 (QCM sensor). Based on these data S/N ratios obtained with SAW resonators are comparable with those obtained with QCM sensors or may also be higher, depending on the protein.

**Table 2:** Frequency shifts Δ*f* obtained by adsorption of HSA and fibrinogen, *c* = 250 µg/mL, in PBS on parylene C coated SAW resonators (*f*_0_ = 426.4 MHz) and QCM sensors (*f*_0_ = 4.95 MHz). Each protein was tested three times using a separate sensor for each experiment. The noise amplitudes were determined in the respective time interval prior to fibrinogen adsorption.

Adsorbed Protein	SAW Resonator Difference Frequency Shift		QCM Sensor Frequency Shift	
	Δ*f* [kHz]	Δ*f*/*f*_0_ [ppm]	Δ*f* [Hz]	Δ*f*/*f*_0_ [ppm]
HSA	12.6 ± 2.3	29.5 ± 5.3	−54.2 ± 2.3	−10.9 ± 0.5
Fibrinogen	121.9 ± 11.9	285.9 ± 27.9	−126.7 ± 2.4	−25.6 ± 0.5
None	Noise: 0.08 ± 0.02	Noise: 0.19 ± 0.04	Noise: 0.32 ± 0.06	Noise: 0.06 ± 0.01

As depicted above, HSA and fibrinogen adsorption on parylene C coated sensors resulted in increasing difference frequencies of the SAW resonators and decreasing frequencies of the QCM sensors. Both increase and decrease came to a stop until a plateau was reached, which level was maintained till the end of the respective sampling interval. Though the same protein concentration was applied as for HSA adsorption, frequency shifts obtained with fibrinogen were higher (according to amount) than frequency shifts obtained with HSA. At a first glance this is in accordance with the higher molecular weight of fibrinogen (MW 340 kDa) compared to HSA (MW 66 kDa). However, while the molecular weight ratio MW(fibrinogen) : MW(HSA) is 5.15, the ratio of the respective SAW resonator signals is 9.67 and the ratio of the QCM sensor signals is 2.34, which means that none of the signal ratios represent the molecular weight ratio. Instead, the SAW resonator signal ratio is higher, and the QCM sensor signal ratio is lower than the molecular weight ratio. It comes as no surprise that the frequency ratio of the acoustic sensors is different from the molecular weight ratio, because the resonance frequency is influenced by both the protein mass (including water) and the viscoelasticity of the adsorbed protein layer. The viscoelasticity in turn is influenced, among others, by the protein conformation (depending on size and shape) and orientation on the surface. As the fibrinogen molecule is larger than the HSA molecule and elongated instead of almost globular, it is obvious that different packaging densities and, hence, viscoelastic properties of the protein layers are obtained, as confirmed by dissipation monitoring (Figure 7b). The influence of the viscoelasticity on the acoustic sensor signal depends on the type of the acoustic sensor. However, to explain the wide discrepancy between the signal ratios fibrinogen: HSA of SAW resonator and QCM sensor, an additional parameter has to be taken into account, *i.e.*, changes in the electrical environment influencing the electromechanical coupling. This effect can effectively be reduced in QCM sensor setups [32], but it has a high impact on the SAW resonator signal response [47]. Dissolving the protein to be investigated in the same buffer as used as carrier medium, as done in this work, helps to minimize such effects if they arise from the sample background. At physiological conditions, however, *i.e.*, pH 7.4 and ionic strength 0.15 M (KCl/NaCl) as used in this work, different net charges have been predicted for the proteins: −19 elementary charges for HSA (isoelectric point: 4.7) [48,49] and −8 elementary charges for fibrinogen (isoelectric point: 5.8) [17]. Hence, differences in mass and viscoelastic properties of the adsorbed protein layers are combined with differences in the electrical environment of the layers, as suggested by the SAW resonator measurements. Consequently, the use of both QCM sensor and SAW resonator allows a more comprehensive characterization of the adsorbed layer than the use of one of these acoustic sensors alone.

**Figure 8:**
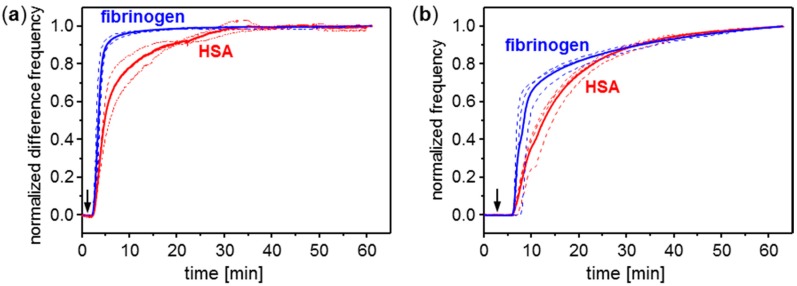
Conditioning film monitoring with (**a**) SAW resonators and (**b**) QCM sensors coated with parylene C: normalized frequency curves (dashed lines) obtained by adsorption of plasma proteins HSA (red curves) and fibrinogen (blue curves). Solid lines represent average curves. Samples contained 250 µg/mL protein in PBS. The PBS carrier stream was switched to sample solution (a) 1 min and (b) 3 min after start of the measurement (see arrows).

To allow for a better comparison of how HSA and fibrinogen adsorption rates are visualized by SAW resonator and QCM sensor measurements, frequencies were normalized to the respective maximum values. Figure 8 summarizes the frequencies obtained during the first hour of injection, normalized to the respective values obtained at the end of this interval. 

Both SAW resonator and QCM sensor measurements show significantly steeper normalized frequency curves for fibrinogen (Figure 8, blue curves) than for HSA (Figure 8, red curves), *i.e.*, fibrinogen adsorbs more rapidly than HSA on the parylene C coated surfaces. This is in agreement with earlier investigations on protein adsorption on hydrophobic surfaces [15,23]. Remaining discrepancies between normalized SAW resonator and QCM sensor frequencies result from differences in the fluidic setups and, hence, measurement protocols; details see “Experimental Section”. The SAW resonator measurement setup was realized with reduced tube connections and flow channel dimensions compared to the QCM sensor measurement setup. As a result, SAW resonator signals increased earlier and with a higher slope after start of injection compared to the respective QCM frequency curves. 

### 3.2. Conditioning Film Formation on Polymers: Adsorption of HSA on Parylene C, PMMA, and PS

SAW resonators were coated with the polymers parylene C, PMMA, and PS. Static water contact angles on the polymer coated SAW resonators were determined to verify the quality of the coating (Table 3). The water contact angles obtained with the polymer coatings were in agreement with contact angles published earlier on similar surfaces. Furthermore, all contact angles obtained with the polymer coated SAW resonators were different from the contact angle determined with uncoated resonators. This shows that both the CVD coating (parylene C) and the spin coating (PMMA and PS) processes were applied successfully for polymer coating of the SAW resonators.

**Table 3:** Polymer coated SAW resonators (*f*_0_ = 426.4 MHz): Contact angle with water and difference frequency shifts Δ*f* obtained by HSA adsorption (*n* = 4). Samples contained 1 mg/mL HSA in PBS and were injected into a PBS carrier stream, injection interval: 1–5 min. Difference frequency shifts of the plateau were determined at the end of the injection interval.

Polymer	Contact Angle [°]	Contact Angle [°] in Literature	Difference Frequency Shift by HSA Adsorption	
			Δ*f* [kHz]	Δ*f*/*f*_0_ [ppm]
none	77.9 ± 0.9	n/a	24.2 ± 5.2	57 ± 12
Parylene C	85.4 ± 2.2	85.1 ± 1.2 [50]	23.5 ± 2.8	55 ± 7
PMMA	71.3 ± 1.4	67.8 ± 1.4 [51]	22.3 ± 2.1	52 ± 5
PS	80.4 ± 3.1	80 [52]	22.0 ± 2.6	52 ± 6

Uncoated and polymer coated SAW resonators were used to monitor the adsorption of HSA on the respective surfaces. HSA adsorption led to similar SAW resonator signal shifts within the standard deviation range (Table 3). This is in agreement with the similar water contact angles, which show that the polymers provide similar hydrophobic surfaces, because HSA, as most proteins, adsorbs readily on hydrophobic surfaces [16,18].

## 4. Conclusions

In this work, we investigated the suitability of SAW resonators for proteinaceous conditioning film monitoring. SAW resonators were successfully coated with polymers by CVD and spin coating procedures and could be applied for time-resolved monitoring of plasma protein adsorption. Comparing the results obtained with SAW resonators with results obtained with QCM sensors demonstrated that SAW resonators provide complementary results, which are beneficial for a comprehensive characterization of protein adsorption. Investigation of protein mixtures and real samples with SAW resonators will be performed in the next future. 
